# Identification, visualization and clonal analysis of intestinal stem cells in fish

**DOI:** 10.1242/dev.134098

**Published:** 2016-10-01

**Authors:** Narges Aghaallaei, Franziska Gruhl, Colin Q. Schaefer, Tobias Wernet, Venera Weinhardt, Lázaro Centanin, Felix Loosli, Tilo Baumbach, Joachim Wittbrodt

**Affiliations:** 1 Centre for Organismal Studies (COS), Heidelberg University, 69120Heidelberg, Germany; 2 Laboratory for applications of synchrotron radiation, Karslruhe Institute for Technology (KIT), 76131Karlsruhe, Germany

**Keywords:** Medaka, Cell division mode, Digestive tract, Intestinal stem cells

## Abstract

Recently, a stochastic model of symmetrical stem cell division followed by neutral drift has been proposed for intestinal stem cells (ISCs), which has been suggested to represent the predominant mode of stem cell progression in mammals. In contrast, stem cells in the retina of teleost fish show an asymmetric division mode. To address whether the mode of stem cell division follows phylogenetic or ontogenetic routes, we analysed the entire gastrointestinal tract of the teleost medaka (*Oryzias latipes*). X-ray microcomputed tomography shows a correlation of 3D topography with the functional domains. Analysis of ISCs in proliferation assays and via genetically encoded lineage tracing highlights a stem cell niche in the furrow between the long intestinal folds that is functionally equivalent to mammalian intestinal crypts. Stem cells in this compartment are characterized by the expression of homologs of mammalian ISC markers – *sox9*, *axin2* and *lgr5* – emphasizing the evolutionary conservation of the Wnt pathway components in the stem cell niche of the intestine. The stochastic, sparse initial labelling of ISCs ultimately resulted in extended labelled or unlabelled domains originating from single stem cells in the furrow niche, contributing to both homeostasis and growth. Thus, different modes of stem cell division co-evolved within one organism, and in the absence of physical isolation in crypts, ISCs contribute to homeostatic growth.

## Abstract (precis)

**Summary:** Adult medaka intestinal stem cells (ISCs) proliferate within a niche functionally equivalent to that in the mammal. Like mammalian ISCs, but unlike medaka retinal stem cells, their mode of division is largely symmetric.

## INTRODUCTION

Recent key findings have stimulated the debate about the division mode of adult stem cells (symmetric versus asymmetric) in mammals in particular and vertebrates in general. While on the one hand prominent symmetric cell divisions followed by neutral drift are best at explaining the division of multipotent intestinal stem cells (ISCs) in mouse (Snippert et al., 2010), the preferential asymmetric division mode of multipotent stem cells drives functional homeostasis during retinal growth in the teleost (Centanin et al., 2014). This poses the question as to whether there is an ancestral (asymmetric) and derived (symmetric) mode of stem cell progression. Alternatively, and independent of the phylogenetic position of the organism, stem cells could divide by symmetric or asymmetric division in a niche-specific manner. The answer may shed light on the interplay between the presence of stem cells and the formation of tumours. Adult stem cells with self-renewing properties (e.g. ISCs) are crucial for tissue homeostasis and closely resemble the properties of tumour cells. The well-described, predominant asymmetric division mode of teleost retinal stem cells allows us to compare how stem cells behave under conditions of organ growth and tissue homeostasis in the life-long growing intestine.

The basic vertebrate bauplan of the digestive tract in fish shows adaptations related to phylogeny, ontogeny, environment and diet of each species (Grosell et al., 2010). Along the rostro-caudal axis, it is divided into three segments: the rostral intestinal bulb, the mid-intestine and the caudal intestine (Wallace et al., 2005; Wang et al., 2010). Microarray analyses of the expression of metabolic genes in the adult zebrafish gut suggest subdivision into small and large intestine (Wang et al., 2010). Similar to the mammalian intestine, the inner layer of the intestine is folded in many fish species, although intestinal folds are often less distinct; however, the villi and crypts found in mammals are not described for medaka or zebrafish.

In mammals, ISCs drive homeostasis by the continuous replacement of the intestinal epithelium. This renewal is species specific and takes between two and seven days (Clevers, 2013a; Creamer et al., 1961; Crosnier et al., 2006). Gene targeting and lineage tracing studies in mouse revealed that ISCs residing in the intestinal crypt base of the small intestine divide and the descendants are pushed towards the villus tip where they exfoliate (Barker et al., 2007; Clevers, 2013b; Ritsma et al., 2014; Tan and Barker, 2014; Tian et al., 2011; Vermeulen and Snippert, 2014). The Wnt pathway plays a key role in intestinal homeostasis and for the maintenance of ISCs (Clevers et al., 2014; Fevr et al., 2007; Korinek et al., 1998). Among the Wnt target genes, Lgr5 is, to date, the most important marker for stem cells of the small intestine and colon (Barker et al., 2007). Lineage tracing experiments in mouse revealed that ISCs expressing *Lgr5* or *Bmi1* can repopulate entire intestinal crypts (Barker et al., 2007; Sangiorgi and Capecchi, 2008). The high mobility group box transcription factor Sox9 is another Wnt target gene regulating cell proliferation in the intestine (Bastide et al., 2007; Blache et al., 2004). Its loss of function affects differentiation throughout the intestinal epithelium and results in the loss of Paneth cells (Bastide et al., 2007), which provide important niche factors to keep ISCs in their proliferative state (Sato et al., 2011).

In the lifelong growing fish intestine, a domain of proliferating epithelial cells was reported at the base of the intestinal folds (Rombout et al., 1984; Stroband and Debets, 1978; Wallace et al., 2005), but the molecular setup of these epithelial cells has not been addressed so far. To compare the mode of stem cell division in the growing retina with stem cell division during homeostasis and tissue growth in the intestine of medaka, we analysed the intestine by high-resolution X-ray microcomputed tomography (microCT), histochemistry and gene expression studies and the characterization of ISCs with molecular, genetic and lineaging tools. We show key morphological and molecular features such as the division into a large and small intestine, the presence of folds and the distribution of proliferative and apoptotic cells along the folds of the medaka intestine. Importantly, we identify a proliferative compartment in the furrows between the intestinal folds that in many respects resembles the mammalian stem cell niche in the intestinal crypts. These cells express homologs of mammalian ISC markers, including *lgr5*, which has not been previously reported in teleosts. Our lineage analysis data are consistent with a predominantly symmetric mode of stem cell division in the intestinal furrows of the medaka intestine, which results in homeostatic growth. Taken together, our data indicate that the mode of division of particular stem cells is not a species-­specific feature but is rather a feature of the specific stem cell type that is correlated with the function of the stem cells in tissue homeostasis and/or organ growth.

## RESULTS AND DISCUSSION

### Morphology of the medaka gut

**Fig. 1.**
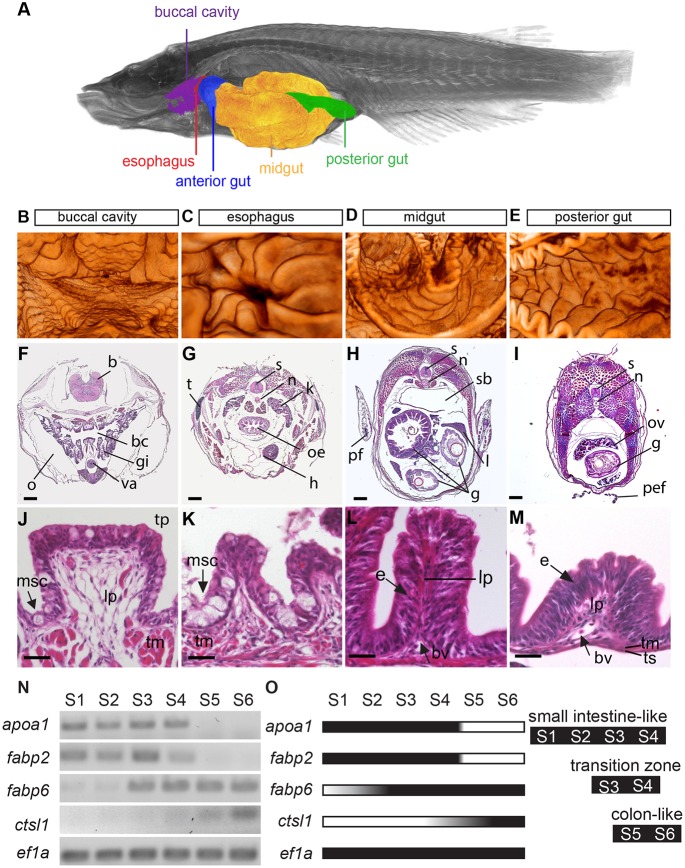
**Medaka intestinal tract shows morphological and functional homology to** **mammalian intestine.** (A) 3D image of adult medaka taken by X-ray microCT. Anatomical landmarks are highlighted. Data were used for reconstruction of the buccal cavity (B), esophagus (C) (rostral to caudal perspective in B,C), midgut (D; anterior: left with densely packed folds; posterior: right with elongated folds), posterior gut (E; anterior: left; posterior: right). (F-I) H&E stained transverse sections of adult gut along rostro-caudal axis. Histology of intestinal folds in each segment is shown below in J-M. Morphology of folds varies along rostro-caudal axis. (N) Gene expression of selected marker genes in six rostro-caudal segments of adult intestine. Control: elongation factor 1α. Note that *apoa1* and *fabp2* are only detectable in four rostral segments. Expression of large intestinal marker *fabp6* is confined to caudal segments S3 to S6 and *ctsl1* to segments S5, S6. (O) Schematic summary of RT-PCR results. b, brain; bc, buccal cavity; bv, blood vessel; e, enterocyte; g, gut; gi, gills; h, heart; l, liver; lp, lamina propria; msc, mucous-secreting goblet cells; n, notochord; o, operculum; oe, oesophagus; ov, ovary; pef, pelvic fin; pf, pectoral fin; sb, swim bladder; s, spinal cord; t, thymus; tm, tunica muscularis; tp, tongue papilla-like; ts, tunica serosa; va, ventral aorta. Scale bars: 200 µm for F-I and 25 µm for J-M.

To characterize the overall morphology of the adult medaka gut with subcellular resolution, we used a microCT station at the TOPO/TOMO beamline (Rack et al., 2009). X-rays penetrate thick and opaque tissues to provide a complete three-dimensional image of large samples *in toto* without the need for sectioning. We recorded and segmented an *in toto* perspective of the gut of a young adult medaka. This 3D view reveals three distinct topographic domains along the rosto-caudal axis of the intestinal tract: the buccal cavity (mouth), the oesophagus and the intestine, the latter characterized by varying shapes from anterior to posterior (Fig. 1A; Movies 1 and 2). We noticed a marked difference in the cavity of the anterior intestine in comparison to the posterior intestine. The bile duct, connecting the gall bladder with the anterior part of the intestine (ductus choledocus, Fig. S1A) marks a position equivalent to the duodenum in mammals. The inner wall of the gut in medaka is wrinkled into structures protruding into the lumen (folds). The lumen size and the density and extent of folds are decreasing along the rosto-caudal axis (Fig. 1B-E).

To assess the morphology of the epithelium in higher detail, we applied Haematoxylin & Eosin staining to histological transverse-sections of 7-week-old fish. The buccal cavity contains papillae, formed by high prismatic epithelial cells containing a large number of the mucous-secreting goblet cells (Fig. 1F,J). The oesophageal mucosa is folded into ridges that are strongly surrounded by muscles (Fig. S1B,C). The epithelium is stratified with numerous intraepithelial aggregates of mucous-secreting goblet cells (Fig. S1D,E). This high number of mucous-secreting cells facilitates the flow of food towards the intestine. The prismatic cells that form the intestinal epithelium rest on connective tissue containing blood vessels and muscle fibres, similar to the lamina propria of the mammalian intestine (Fig. 1J-M).

Folds in the anterior intestine and the midgut are densely packed and display an elongated, ridge-like shape (Fig. 1H,L). The number of folds decreases towards the tail: they broaden, get shorter and are almost absent close to the anus (Fig. 1F-M). In mammals, ISCs reside in crypts of Lieberkühn of the intestine. We did not identify analogous invaginations in the medaka intestine. Columnar-shaped enterocytes are the most prominent cell type of the intestinal epithelium followed by the mucous-secreting goblet cells, present in all intestinal domains (Fig. 1J-M). Altogether, our morphological analyses show that the medaka and mammalian digestive tract share a number of features.

### Subdivision of the medaka gut into a small and large intestine

To examine whether the morphological domains of the intestine correlate with distinct gene expression domains, we analysed specific marker genes for the small and large intestine present in the respective structures of the mammalian intestine. We divided the gut of a young adult medaka (buccal cavity and oesophagus were not included) into six segments (S1 to S6) and addressed the expression of the intestinal marker genes in each segment by semi-quantitative RT-PCR. As markers for the small intestine, we detected the apolipoprotein *apoa1* as well as the fatty acid binding proteins *fabp2* and *fabp6*. Apoa1 is a key player in cholesterol homeostasis and is highly restricted to the digestive organs including the small intestine (Wang et al., 2010). Consistently, medaka *apoa1* was detected in the first four gut segments (Fig. 1N-O). In mammals, Fabp2 is involved in the uptake and intracellular transport of fatty acids in the small intestine (Chmurzynska, 2006). Like *apoa1*, medaka *fabp2* was expressed only in the first four gut segments (S1-S4). In mammals, Fabp6 acts as an intracellular transporter of bile acids in ileal epithelial cells (most distal part of the small intestine), helping to catalyse and to metabolize cholesterol (Praslickova et al., 2012). In medaka, *fabp6* expression was detected in segments S3-S6, with a very weak expression in S1 and S2 (Fig. 1N,O). Cathepsin L1 (*ctsl1*) is a marker for the colon-like intestinal region in zebrafish (Wang et al., 2010) and is found in the corresponding domain in medaka (S5 and S6).

The expression profiles of the marker genes, as well as the histological features of the medaka intestine, indicate that segments S1-S4 of the medaka intestine resemble the mammalian small intestine. This stretch comprises long and densely packed folds and exhibits, with the expression of *apoa1* and *fabp2*, a fingerprint comparable to the mammalian small intestine. The low expression of *ctsl1* as well as expression of *fabp6* in segments S3/S4 indicates a distinct transition zone between small and large intestine. The remaining segments S5 and S6 of the medaka intestine have large intestine characteristics, as indicated by broader and fewer folds and the strong presence of the colon markers *ctsl1* and *fabp6*.

### Expression of ISC markers in the intestinal furrows

**Fig. 2.**
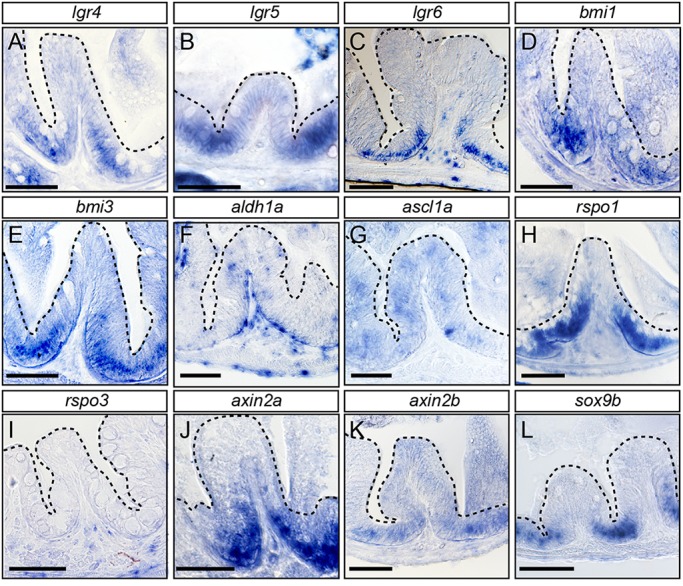
**Stem cell marker genes** **are** **expressed at the base of intestinal folds in the adult intestine.** RNA *in situ* hybridization of markers on sections of the medaka adult intestine, anterior and midgut. (A-C) Graded expression of *lgr4*, *lgr5* and *lgr6* from base to centre of intestinal folds. (D-F) Weak *bmi1*, *bmi3* and *aldh1a* expression, with more prominent *aldh1a* in *lamina propria* and supporting tissues. (G) *ascl1a* expression restricted to patches. (H,I) *rspo1* is expressed within fold, whereas *rspo3* is confined to underlying connective tissue. (J-L) *axin* (*axin2a* and *axin2b*) and *sox9b* show clear expression confined to the base of the fold. Dashed lines delineate intestinal folds. Scale bars: 50 µm.

The high morphological similarity between the medaka and the mammalian intestine probably extends into the molecular mechanisms controlling ISCs. In the murine intestine, several signalling pathways, including Wnt, Notch, BMP (bone morphogenetic protein) and Hedgehog orchestrate epithelial homeostasis (Sancho et al., 2004). Lgr5, a member of the family of G-protein-coupled receptors, represents a Wnt target gene and is a prominent marker for ISCs residing at the base of the crypts (Baker et al., 2015; Barker et al., 2007; Clevers et al., 2014). While *lgr4* and *lgr6* have been described in zebrafish (Hirose et al., 2011) and medaka (Deguchi et al., 2012), there are no reports of *lgr5* expression in fish. We identified the orthologue of *lgr5* in the medaka genome and analysed its expression together with the expression of additional ISC marker genes in the intestine of young adult medaka (Fig. 2). We found that the expression of *lgr5* and its paralogs *lgr4* and *lgr6* is confined to the base of the intestinal furrows in the medaka small intestine (Fig. 2A-C).

We used expression of the polycomb complex protein *bmi1*, a marker for quiescent ISCs in mouse (Sangiorgi and Capecchi, 2008; Yan et al., 2012), to identify the equivalent cells in medaka. Here, a weak expression of *bmi1* and *bmi3* was detected at the base and the mid-base segments (Fig. 2D,E).

In addition, we investigated the expression of several other marker genes specific for ISCs or sub-structures of the mammalian intestinal crypt. We detected expression of the aldehyde dehydrogenase *aldh1a*, a marker for normal and cancer stem cells (Huang et al., 2009) in the connective tissue underlying the intestinal furrows (Fig. 2F).

The gene *ascl1a* (transcription factor achaete scute-like), an orthologue of the mammalian transcription factor and intestinal stem cell marker Ascl1 (Rizk and Barker, 2012; van der Flier et al., 2009), is expressed throughout the entire fold with sporadic elevated levels in single cells in the intestinal furrow (Fig. 2G). R-spondins enhance low-dose Wnt signals (Kazanskaya et al., 2004) and Rspo1 stimulates crypt stem cell proliferation in mouse (Kim et al., 2005). We detected expression of the medaka *rspo1*at the base of the intestinal furrow (Fig. 2H,I). *Rspo3* is not expressed in the fold itself, but in cells of the underlying connective tissue similar to *aldh1a* (Fig. 2I). Axin is an important Wnt suppressor (Ikeda et al., 1998) and the medaka paralogs *axin2a* and *axin2b* were detected in intestinal epithelial cells, where *axin2b* was more restricted to the base of the intestinal furrow (Fig. 2J,K).

Of all marker genes highlighting ISCs, the transcription factor Sox9 showed the most confined expression pattern. In mouse, distinct levels of *Sox9* are expressed in stem and differentiated cell populations of the small intestinal epithelium (Formeister et al., 2009; Furuyama et al., 2011; Gracz et al., 2010; Van Landeghem et al., 2012). Schartl and colleagues identified *Sox9* paralogs (*sox9a*, *sox9b*) in teleosts (Kluver et al., 2005) and show that only *sox9b* is expressed in the distal intestine of late embryos and early juveniles. In our analysis of the adult medaka intestine, *sox9b* expression is confined to a small number of cells at the base of the intestinal furrow (Fig. 2L). Taken together, the consistent localized expression of ISC marker genes at the intestinal furrow of the adult medaka intestine suggests that this domain harbours ISCs in medaka. Our analysis is consistent with the idea that the molecular mechanisms underlying ISC proliferation in fish resemble those proposed for mouse.

### Sox9b is a putative marker for proliferative progenitor and ISCs in medaka

**Fig. 3.**
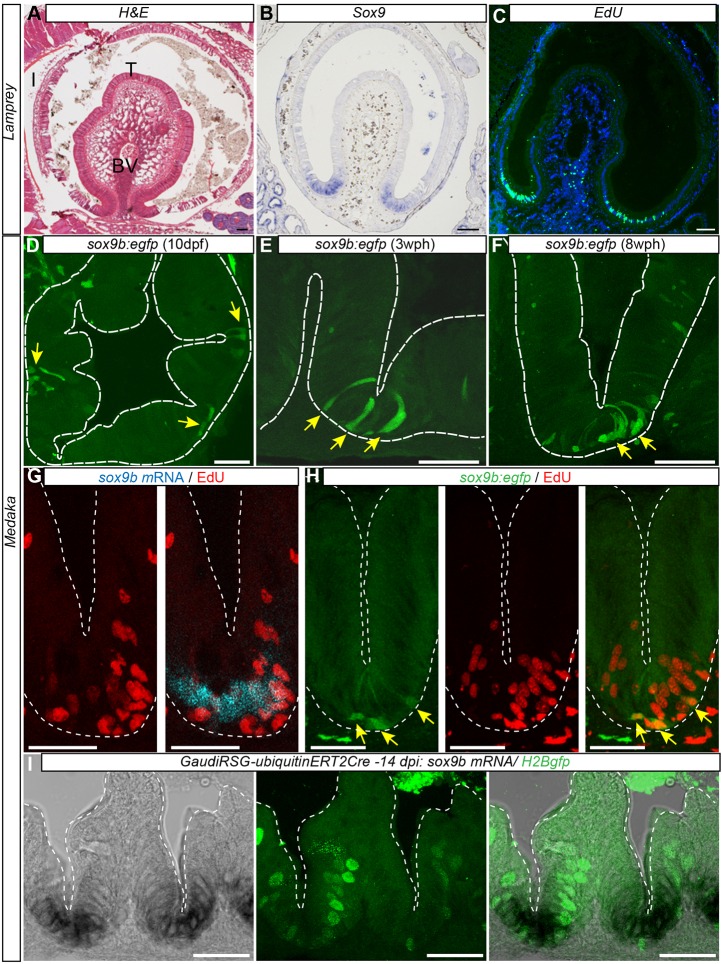
**Expression pattern of intestinal *sox9* is conserved from lamprey to medaka.** (A) Transverse section of lamprey larval intestine stained with H&E, highlighting the typhlosole as a single fold. (B) *In situ* detection of *Sox9* expression at the base of the typhlosole. (C) EdU^+^ cells (24 h after injection) reveal basal proliferation zone (green; nuclei: DAPI, blue). (D-F) Medaka *Sox9b:eGFP*-expressing cells mark proliferative intestinal cells at the base of furrows. Confocal images of transverse cryosections of *sox9b:gfp* transgenic intestine at 10 dpf (D) in 3-week-old juveniles (E) and 8-week-old adult fish (F). Arrows indicate position of *sox9b:eGFP*-expressing cells at the base of folds. (G) Colocalization of endogenous *sox9b* expression domain shown by *in situ* hybridization and proliferation in EdU^+^ cells. (H) Colocalization of *sox9b:eGFP*-expressing cells and EdU staining. Arrows indicate *sox9b:eGFP*-expressing cells. Note that *sox9b:eGFP*-expressing cells are EdU^+^. (I) *Sox9b* endogenous expression shown by *in situ* hybridization in Gaudí*^RSG^*-*ubiquitin:^ERT2^Cre* transgenic fish (left panel), clonal analysis of Gaudí*^RSG^*-*ubiquitin:^ERT2^Cre* transgenic fish 2 weeks after induction. At the base of the furrow, proliferating GFP^+^ stem cells (middle panel) express *sox9b* (right panel). Scale bars: 100 µm (A-C); 25 µm (D,G,H,I); 50 µm (E,F). BV, blood vessel; I, intestine; T, typhlosole.

The transcription factor Sox9 is required for the induction and maintenance of several types of vertebrate stem cells including ISCs (Formeister et al., 2009; Furuyama et al., 2011; Gracz et al., 2010; Van Landeghem et al., 2012). The conspicuous expression of *sox9* in different models including *Xenopus* (Lee and Saint-Jeannet, 2003), chicken (Shyer et al., 2015) and mouse (Blache et al., 2004) hints at an essential role of this gene in the gut. In the larval gut of the basal vertebrate lamprey (*Lampetra planeria*), we identified *sox9* expression in proliferating cells (described previously by Bajoghli et al., 2011) at the base of the typhlosole (Fig. 3A,B), a longitudinal fold of the inner intestinal wall (Fig. 3C), hinting at its evolutionarily conserved function (Fig. 3).

In medaka, expression of the mammalian *sox9* orthologue (*sox9b*) was reported in the gonads (Kluver et al., 2005; Nakamura et al., 2010). We show that *sox9b* is also expressed in the intestine, confined to the furrow between the intestinal folds. To correlate expression and proliferative capacity of sox9b^+^ cells, we used transgenic fish expressing eGFP under the control of the *sox9b* regulatory elements (Nakamura et al., 2008). Here, eGFP expression is restricted to a few intestinal epithelial cells located at the furrow between the intestinal folds at larval stages (10 days post fertilization, dpf), 3-week-old juvenile and 8-week-old adult fish (Fig. 3D-F).

To test whether cells expressing *sox9b* actively proliferate, we assayed EdU incorporation in sox9b^+^ cells and co-detected EdU and *sox9b* by whole-mount *in situ* hybridization or in the transgenic line. Twelve hours after a 24 h EdU pulse, sox9b^+^ cells colocalize with EdU^+^ cells (Fig. 3G) in young adults. We conducted a comparable EdU pulse-chase experiment in 1-month-old fish in the *sox9b::eGFP* transgenic line. In those fish, cells expressing *sox9b* also incorporated EdU 24 h after the EdU pulse (Fig. 3H, *n*=3 fish, 63 cells), indicating the proliferative activity sox9b^+^ cells in the juvenile and adult medaka intestine. Finally, to address the stem cell nature of sox9b^+^ cells, we detected Sox9b expression initiator cells in a clonal lineage. We analysed *sox9b* in the Gaudí transgenic line (Centanin et al., 2014) initiating lineaging by stochastic activation of *ubiquitin*:*^ERT2^Cre* in Gaudí*^RSG^* fish by tamoxifen treatment (10 µM, 3 h) at stage 40 of development. Two weeks after induction, we correlated lineage traces and *sox9b* transcripts by confocal microscopy (Fig. 3I). All GFP^+^ lineages analysed (*n*=66 lineages in 4 fish) colocalized with *sox9b* expression at their point of origin in the proliferative zone at the base of the intestinal furrows. Thus, *Sox9b* represents an evolutionarily conserved marker for proliferative progenitors and ISCs.

### Proliferative cells are predominantly located in furrows between intestinal folds

**Fig. 4.**
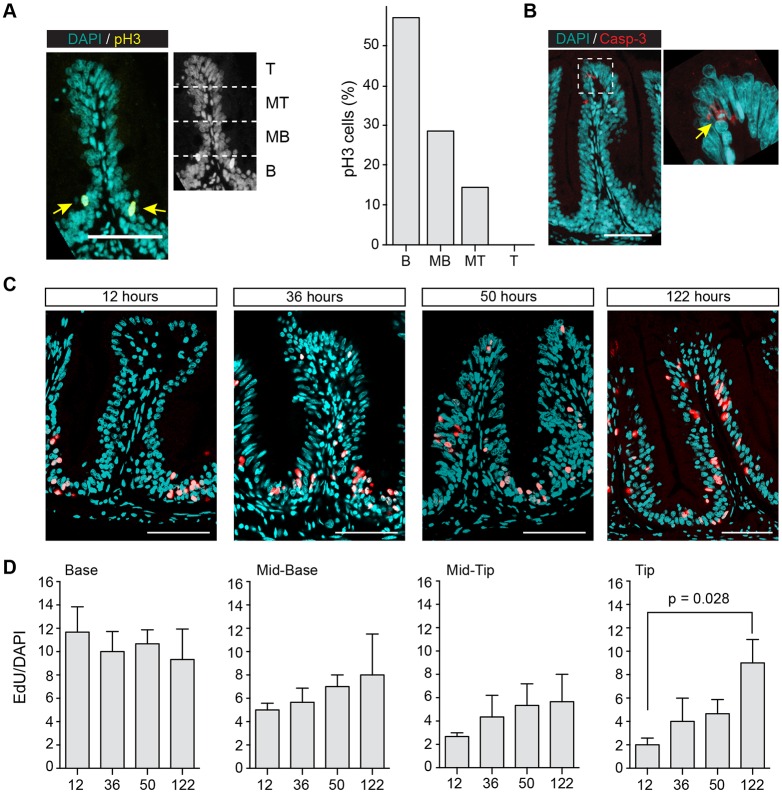
**Intestinal stem cells** **are** **located in furrows between intestinal folds.** (A) pH3 immunostaining on transverse sections of fold. Fold was divided into four equally sized regions: base (B), mid-base (MB), mid-tip (MT) and tip (T). Arrows in left panel indicate pH3^+^ cells. Respective frequency of pH3^+^ cells is shown in right panel. The majority of pH3^+^ cells are seen at the base of the fold. (B) Caspase-3 immunostaining on transverse sections of a fold. Caspase-3^+^ cells located at fold tip. (C,D) EdU pulse-chase assay. Adult fish were incubated in EdU for 24 h and fixed after 12, 36, 50, 122 h. For each time point, a representative fold from anterior and mid gut is shown in C. (D) Frequency of EdU^+^ cells in each region was analysed with KNIME. For each time point, three fish were analysed and nuclei from around 50 folds were counted for each region. Values are mean±s.e.m. A significant difference between 122 and 12 hours in the tip region was found using unpaired Student's *t*-test; *P*=0.028. Scale bars: 50 µm.

It has been proposed in previous studies in different fish species that compartments of proliferating epithelial cells are located at the base of the intestinal folds (Faro et al., 2009; Hellberg and Bjerkas, 2000; Rombout et al., 1984; Wallace et al., 2005). To identify the position of proliferating cells and ISCs in the medaka gut, we stained mitotically active cells immunohistochemically detecting phosphorylated histone H3 (pH3) and EdU incorporation. We subdivided the intestinal folds from basal to apical into four equally sized segments: base (B), mid-base (MB), mid-tip (MT) and tip (T) and determined the relative number of pH3^+^ and EdU^+^ cells in each of the segments (Fig. 4A). Analysis of cells in M-phase revealed that the majority of pH3^+^ cells (85.71%, *n*_Folds_=20) is located in the basal half of the folds (B+MB). Only few proliferating cells were found in MT (14.9%, *n*_Folds_ =20) and none in T (Fig. 4A). We resolved the temporal dynamics of intestinal proliferation in an EdU pulse-chase assay. Young adult fish were incubated in EdU for 24 h and were sacrificed 12, 36, 50 and 122 h post-treatment. The number of EdU^+^ cells was counted in each segment of the folds at the time point indicated (Fig. 4C). This revealed a progression of initially labelled cells from the basal sector to the tip of the folds. The number of EdU^+^ cells remained always highest at the base of the fold, consistent with the stem cell niche being located in the furrow between the folds. The increase of EdU^+^ cells at the tip (T) from 4.6±1.5% at 12 h post-incubation to 14.0±4.3% at 120 h post-incubation (Fig. 4D) reflects the flux of cells from base to tip. Apoptotic cells only present at the tip of the fold indicates the shedding of intestinal cells in this segment (Fig. 4B). Similar experiments in zebrafish had determined a turnover time of 7-10 days for the base to tip transition (Wallace et al., 2005).

Taken together, our results identify a population of mitotically active cells located at the base between intestinal folds. EdU^+^ cells are initially found at the base and are subsequently shifted upwards to the tip. Our data in medaka indicate that it takes 5-10 days from the birth of intestinal cell types at the base to apoptosis at the fold summit, where cells are shed off into the intestinal lumen.

### Identification of ISCs in the furrow between the intestinal folds

**Fig. 5.**
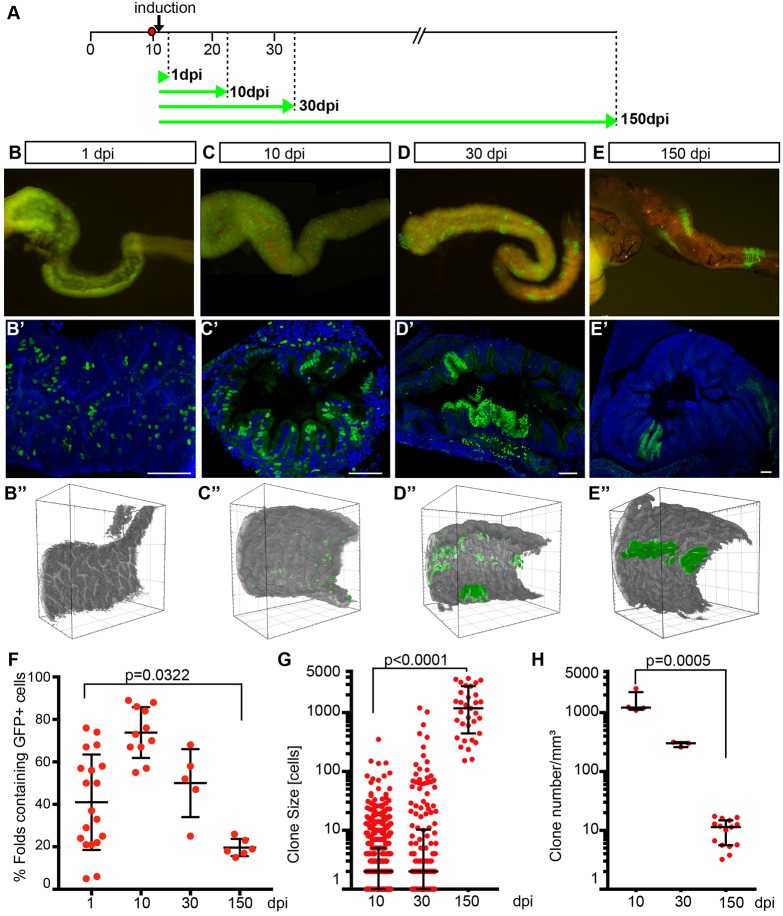
**Clonal analysis in the Gaudí*^RSG^* line** **indicates symmetric** **cell division in the medaka intestine.** (A) Experimental timeline. Double transgenic fish Gaudí*^RSG^*-*ubiquitin:^ERT2^Cre* were used with an inducible Cre recombinase that triggers a shift in reporter colour and localization. Cre-mediated recombination of Gaudí*^RSG^* construct was triggered at larval stage by tamoxifen treatment (5-10 µM, 3 h). Green arrows indicate time point of imaging and analysis of the intestine after induction. (B) Whole mount representation (macroscope) of dissected intestines at different time points (B) 1 day (C) 10 days (D) 30 days (E) 150 days post induction. (B′-E′) Confocal images of intestinal sections of corresponding fish showing labelling of discrete, single cells at 1 dpi, larger clonal strings extending from bottom to top of folds (10 dpi) and coverage of entire folds with descendants of individual recombined (or non-recombined, 30 dpi, 150 dpi) cells. Each panel represents a 3D projection of 60-100 optical sections (plane=0.5 µm). H2B-GFP, green; DAPI-stained DNA, blue. Scale bars: 50 µm. (B″-E″) MuVi-Spim 3D visualization of gut segments (532 µm) at different time points after induction showing labelling in the context of the organ. (F) GFP^+^ folds were counted using sections from 19 fish at 1 dpi, 11 fish at 10 dpi, 5 fish at 30 dpi, 6 fish at 150 dpi; unpaired *t*-test, *P*=0.0322. (G) Quantification of clone size of intestinal segments shown in B″-E″ at 10, 30, 150 dpi; data are represented at logarithmic scale; Mann–Whitney test, *P*<0.0001. (H) Clone density per volume (mm^3^) of intestinal segments shown in B″-E″. Cell numbers were derived from light sheet analyses and are represented at logarithmic scale; Mann–Whitney test, *P*=0.0005. Black bars indicate mean±s.d. in F and interquartile range in G,H.

To ultimately identify and trace ISCs and their division mode, we performed long-term lineage analysis in the medaka intestinal folds during life-long organ growth and homeostasis. This not only allowed us to follow the lineage of individual cells, but also addressed the mode of stem cell division in the niche. To stochastically label individual cells in the medaka intestine, we used the brainbow-based (Livet et al., 2007) Gaudí toolkit (Centanin et al., 2014). We combined the two-colour fluorescent reporter line Gaudí*^RSG^* (Gaudí red-switch-green) with *ubiquitin:^ERT2^Cre*, a line that allows stochastic activation of Cre recombinase by tamoxifen induction. The timeline of the experiment is presented in Fig. 5A. Tamoxifen applied at late embryonic stages (d12, Fig. 5A) triggers the stochastic recombination in individual cells in the intestine of the Gaudí*^RSG^* line, resulting in a switch from red to nuclear green in recombined cells and all of their descendants. Tamoxifen concentration (5-10 µM) and duration of the treatment (3 h) were adjusted to achieve a labelling of clearly discernible, individual cells within the intestine. After stochastically triggering permanent H2B-EGFP expression, eventually only mitotically active ISCs will contribute to continuous strings of labelled cells. Only if the initial recombination occurred within a stem cell will it retain its label and transmit it to all of its descendants. Conversely, recombination in progenitor or differentiated cells will result in the transient labelling of small clones or individual cells. The replacement of epithelial cells in the intestine will ultimately permit the detection of only labelled ISCs and their clonally continuous descendants (Fig. 5B-E″). In a scenario of predominantly asymmetric stem cell divisions, the total number of clonal strings originating from a single stem cell will remain constant. Symmetric division and neutral drift, conversely, will result in a marked decrease of the number of stem cell clones on the one hand, while at the same time, the clone size will increase.

We investigated the development of stem cell clones over time in three complementary approaches (Fig. 5B-E″) and quantified the density, distribution and number of labelled cells/clones (Fig. 5F-H) in two independent experimental settings with 5 to 20 individuals per time point analysed.

Imaging whole mount preparations of dissected intestines at different time points after clonal induction provides a qualitative impression of the distribution and development of the labelled cells (Fig. 5B-E). The initial labelling of many discrete clones composed of individual cells on the first day post induction (dpi) (Fig. 5B) is strongly reduced over time (Fig. 5C,D) and is eventually (150 dpi) confined to a few very large clones (Fig. 5E). We analysed intestinal sections (30-50 µm) of the same fish by confocal microscopy (Fig. 5B′-E′). This analysis, as shown in a 3D representation of the cross sections, confirms the gross morphological analysis in Fig. 5B-E. Initially, many small clones are evenly distributed (Fig. 5B′). Eventually only few remaining clones extending from the base to the top of the intestinal folds increase in size along the longitudinal axis of the ridge [visible at 10 dpi (Fig. 5C′) and prominent at 30 and 150 dpi (Fig. 5D′,E′, respectively)].

The distribution of labelled clones was quantified (Fig. 5F) in cross sections of the small intestine (5-19 animals per time point, on average seven distantly spaced sections per animal) by relating the number of GFP containing labelled folds to unlabelled folds in the same section. An initially high range of positive cells distributed at 1 dpi is narrowed down at subsequent time points after induction. The relative distribution per section is strongly reduced from 75% at 10 dpi to 19% at 150 dpi (Fig. 5F).

In a third approach, we imaged and counted labelled cells and clones in a large volume of the intestine (532×532×532 µm) using light sheet imaging of the recombined intestines at identical time points after induction (Fig. 5B″-E″,G,H). This analysis highlighted the longitudinal expansion of the clones (growth along with homeostasis) and unambiguously unveiled the symmetric mode of stem cell division. After the initial stochastic labelling of individual cells (1 dpi, Fig. 5B″), symmetric division and neutral drift established larger, clonally related groups of stem cells (10 dpi, Fig. 5C″, Movie 3) eventually resulting in intestinal folds that are either broadly labelled by H2B-EGFP, or not at all (Fig. 5D″,E″, Movies 4 and 5).

With the full 3D dataset of the MuVi-Spim, we quantified clone number, cell number within individual clones and corresponding clone size at three time points after clone induction (10 dpi=693 clones, 4 intestinal segments; 30 dpi=178 clones, 3 intestinal segments; 150 dpi=35 clones, 15 intestinal segments, Fig. 5G,H). In brief, we determined clone volume by thresholding of nuclear H2B-GFP and determined the number of cells per clone by division by the average nuclear volume (details in Materials and Methods). These analyses show an increase in clone size from one cell to 1600 (on average) cells from 10 dpi to 150 dpi, while at the same time, clone numbers decrease from 1000 to 12 clones per mm^3^ (Fig. 5E′,E″,H, Movie 5). The quantification of label distribution, cell numbers and clonal density underpins the visual impression and strongly supports symmetric cell division of ISCs followed by neutral drift to contribute to homeostatic growth.

Taken together, our qualitative and quantitative analyses revealed a clear prevalence of symmetric stem cell divisions in the medaka intestine, analogous to the findings in the murine intestine and different from the division mode of stem cells in the retina. Symmetric cell division and neutral drift ultimately result in large monoclonal domains of the intestinal epithelium. These domains expand widely in the furrow between the folds (Fig. 5E″, Movie 5) and are eventually composed of hundreds to thousands of stem cells (Fig. 5E,E′,E″,G), contributing to both adjacent folds, arguing for a joint niche for both flanks. Clearly, the intestinal folds physically restrain the stem cells to the furrow. Consequently, each fold is composed of two flanks of separate origin in the respective furrows (Fig. 5D″,E″, Movies 3-5).

**Fig. 6.**
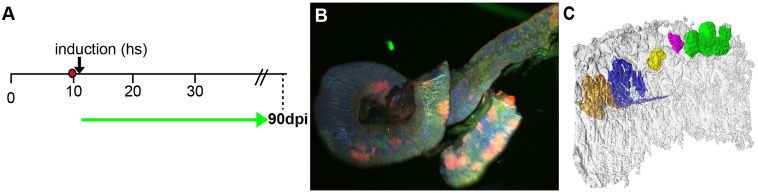
**Clonal cell lineage tracing using Gaudí*^LxBBW^* line confirms mode of cell division in the medaka intestine.** (A) Experimental timeline. Double transgenic fish Hsp70::_nls_CRE were used. Temperature shift inducible Cre recombinase triggers stochastic shift in reporter colour and localization. The shift was triggered at larval stage (12 dpf) and intestines were analysed 90 dpi as indicated by green arrow. (B) Representation of multicolour Gaudí*^LxBBW^* intestinal segment. Note that recombination in the tandem array of the *LxBBW* cassette results in multiple combinations of colours and localization, unambiguously barcoding each individual cell. (C). High-resolution MuVi-Spim visualization (false colour) of intestinal segment (532 µm) showing multi-colour labelling in the context of the organ.

To address whether the large domains form by growth or as a result of the fusion of initial clusters of stem cells, we stochastically triggered clonal multi-colour labelling in the Gaudí*^LxBBW^* line (Centanin et al., 2014) by a limited heat shock activating Cre recombinase via the Hsp70::_nls_CRE transgene (Fig. 6A). We analysed the resulting intestines after 90 days of homeostatic growth by MuVi-Spim imaging and identified adjacent, clonal domains of different sizes, discernible by different fluorescent proteins and their corresponding localization (Fig. 6B,C, Movie 6; *n*=3, eight gut pieces). Taken together all our data consistently argue for individual stem cells undergoing symmetric cell divisions followed by neutral drift, which ultimately results in clonal expansion or shrinking (Figs 5 and 6). In extreme cases, neutral drift can shift entire sections of the fold into a monoclonal valley (Movie 5).

## CONCLUSION

**Fig. 7.**
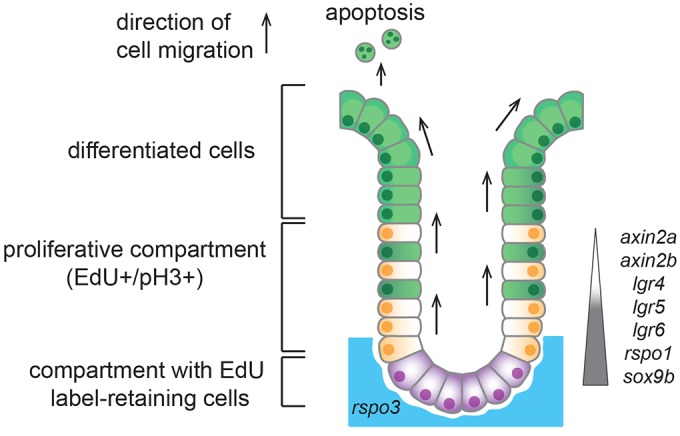
**Model of ISCs in adult medaka fish.** Bulging of the medaka intestinal epithelial surface creates folds and furrows. Proliferatively active intestinal epithelial stem cells are located in furrows at the base of intestinal folds. Apically adjacent, EdU^+^/pH3^+^ domain represents transit-amplifying compartment. Differentiated cells are pushed towards fold tip, where they are shed. *Lgr4/5/6*, *axin2a*, *axin2b* and *rspo1* stem cell markers show graded expression in intestine at base of folds up to mid-base part. *Sox9b* expression is confined to base of folds.

Our detailed study of the fish intestine has revealed a striking similarity to the mammalian intestine ranging from the overall structure to the molecular definition of specific intestinal domains. The entire fish intestine is in continuous growth and homeostasis and all differentiated cell types originating from ISCs are ultimately shed into the intestinal lumen (Fig. 7). ISCs reside in the furrows at the base of intestinal folds and express typical stem cell markers, including lgr5 (Leushacke et al., 2013; Lim et al., 2013), that we discovered in the medaka gut. The comparative analysis of *sox9* (Formeister et al., 2009) expression identifies a domain of proliferatively active cells not only in teleosts, but also in basal vertebrates (lamprey). The expression of *sox9* in proliferating cells at the base of the intestinal fold suggests that the stem cell niche has evolved from a longitudinal, extended furrow in basal vertebrates to an isolated crypt in mammals (Formeister et al., 2009). The multiple folds and furrows in fish represent a transition state. Analysis of the mode of intestinal stem cell division in medaka revealed a predominant symmetric cell division, highly reminiscent of the situation in mammals (Snippert et al., 2010). One of the consequences of the symmetric division mode in mouse is that all intestinal cells in a crypt are eventually of monoclonal origin (Snippert et al., 2010). In the case of the fish intestinal stem cell niche, we uncovered a progressive lateral extension of a ‘monoclonal’ domain, which probably occurs by neutral drift. This domain could, hypothetically, extend along the entire furrow between the intestinal folds. Even though neutral drift controls a monoclonal domain that can grow or shrink, there is no physical barrier to prevent its expansion along the entire intestinal fold. Not restraining longitudinal extension easily facilitates the coupling of tissue homeostasis and organ growth.

The evolution of the intestine and the formation of crypts in birds and mammals ultimately ensure the maintenance of polyclonality by physical isolation – a polyclonality that is composed of individual, monoclonal units represented by the intestinal crypts (Rinkevich et al., 2014; Roy et al., 2014; Shyer et al., 2015). The evolutionary advantage of retaining polyclonality of the intestinal epithelium of mammals and birds goes along with the uncoupling of tissue homeostasis from organ growth, which are still clearly coupled in fish.

The cell division mode in the intestine is in clear contrast to the situation in the continuously growing fish retina, where stem cells predominantly divide asymmetrically to contribute to the growing retina. Therefore, the mode of stem cell division is not a species-specific feature, but is rather characteristic for the type of stem cell niche and the function of the niche in tissue growth and/or homeostasis.

## MATERIALS AND METHODS

### Fish

Medaka (*Oryzias latipes*) stocks were maintained as previously described (Koster et al., 1997). All fish (Icab inbred strain) are maintained as closed stocks at the Centre for Organismal Studies (COS) at Heidelberg University and the Institute of Toxicology and Genetics of the Karlsruhe Institute of Technology (KIT). Fish husbandry and experiments were performed according to local animal welfare standards (Tierschutzgesetz 111, Abs. 1, Nr. 1, Haltungserlaubnis AZ35-9185.64 and AZ35-9185.64/BH KIT) and in accordance with the European Union animal welfare guidelines. The fish facilities are under the supervision of the local representative of the animal welfare agency. Embryos were staged according to Iwamatsu (2004). If not stated otherwise, adult fish were defined as at least 3 months old, which are sexually mature under normal laboratory conditions. The *sox9b*:*EGFP* transgenic fish was described previously (Nakamura et al., 2008).

### X-ray microCT imaging

Seven-week-old medaka were sacrificed, fixed and imaged at the TOPO/TOMO beamline at ANKA, the synchrotron radiation facility of KIT. Fish were sacrificed according to the German animal welfare act and immediately fixed (4% formaldehyde, 1% glutaraldehyde for 3 days at room temperature). For better X-ray contrast, fish were stained with 0.3% phosphotungstic acid in 70% ethanol for 3 days. Samples were washed, embedded in agarose, sealed in polypropylene containers and mounted on a standard tomographic table. We used a parallel, monochromatic beam setup (double-multilayer monochromator, 2% energy bandwidth, energy set to 16 keV) with an additional Al filter (0.2 mm). The optical setup consisted of a LAG scintillator, converting photons to the visible light spectrum, a magnifying microscope setup (total magnification 3.6×) and a CMOS camera (pco.edge, 2560×2160 pixels, 6.5×6.5 µm² pixel size), resulting in an effective pixel size of 1.81 µm. For each sample, we recorded a full 360° tomographic scan of 1500 projections. MATLAB was used for image processing of raw data, unless stated otherwise. PyHST algorithm (Chilingaryan et al., 2011) was used for 3D data reconstruction. Those data were de-noised with a non-local means filter for further analysis. Detailed protocols and scripts are available upon request. General image processing was done with Fiji/ImageJ (Schindelin et al., 2012). Segmentation of the gut was performed by edge-based and region competition snake algorithms with the ITK SNAP software (Yushkevich et al., 2006). 3D rendering for visualization and 3D structure analysis was performed with Amira and ImageSurfer2 (Feng et al., 2007).

### RT-PCR

The gut tubes of three adult medaka females were cut into six equal pieces. Total RNA was extracted from each piece using TRIzol reagent (Ambion) according to the manufacturer's instructions and 1 µg RNA was used to synthetize cDNA with the Superscript First-Strand Reverse Transcriptase kit (Invitrogen) and oligo (dT) primer. Primers for RT-PCR are listed in Table S1. For PCR analysis, the Q5 High-Fidelity DNA polymerase Kit from BioLabs was used. PCR conditions: denaturation 98°C, 30 s; then 30 cycles at 98°C, 10 s; annealing: 57-60°C, 30 s; 72°C, 30 s; final extension 72°C, 2 minutes.

### Isolation of lamprey and medaka probes

*axin2a*, *axin2b*, *rspo1*, *rspo3*, *lgr4*, *lgr5* and *lgr6* genes were amplified from medaka adult gut cDNA. *aldh1a2*, *ascl1a*, *bmi1*, *bmi3*, *sox9b* was isolated from a medaka cDNA library (Souren et al., 2009). Amplified DNA fragments were cloned into the pGEM-T easy vector (Promega) and sequences validated. Primers for PCR and library IDs are listed in Table S2. Linearized DNA was *in vitro* transcribed with Sp6 or T7 polymerase according to the standard protocol for generation of the antisense probes. cDNA of lamprey was provided by the T. Boehm (Max Planck Institute of Immunobiology and Epigenetics, Freiburg, Germany). Primers listed in Table S2 were designed based on the *Petromyzon marinus* Sox9 protein sequence (NCBI GenBank accession number DQ136023.1) and alignment with the Japanese lamprey genome.

### *In situ* hybridization

*In situ* hybridization on paraffin sections was performed with digoxigenin (DIG)-labelled antisense probes as described (Aghaallaei et al., 2005). Images of RNA *in situ* hybridization were taken with a Zeiss Axio Imager.

### EdU proliferation assay

Six-week-old fish were incubated in 100 µM 5-ethynyl-2′-deoxyuridine (EdU) for 24 h and then fixed in 4% paraformaldehyde (PFA)/2× PBST (PBS with 0.1% Tween-20) after 12, 36, 50 and 122 h. After fixation, fish were embedded in paraffin according to standard protocols. EdU detection on sections (7-9 µm) was performed using the Click-iT EdU Alexa Fluor 647 Imaging Kit (Invitrogen) as described previously (Bajoghli et al., 2011). Fluorescence microscopy was performed with a Leica TCS SPE, equipped with a 40× oil objective and AxioVision software. The proliferation assay in lamprey was done as described (Bajoghli et al., 2009) by injection of EdU (5 µg). EdU proliferation assays were performed at EMBL.

### KNIME

Quantification of cell proliferation in the intestine was performed using the KNIME image processing toolbox (KNIP). Segmentation of DAPI-stained nuclei was performed using the KNIP spot detection node. The folds were divided into four equally high trapezoids along the apical-basal axis. Total number of epithelial cells as well as EdU^+^ cells in each of the four trapezoids was determined.

### Lineage tracing, image acquisition and analysis

Medaka Gaudí lines (Centanin et al., 2014) were used for lineaging. Cre recombination with tamoxifen induction (5-10 µM; Sigma, TM5648) was performed for 3 h at 12 dpf (stage 40). For heat shock induction, embryos were incubated at 39°C for 2 h and fixed 90 days post induction (dpi). After 1, 10, 30 or 150 days, fish were sacrificed, fixed in 4%PFA/2× PBST overnight at 6°C, and divided into two groups. One group was cryopreserved in 30% sucrose, embedded in Leica tissue freezing medium and sectioned at 30-50 µm on a cryostat. Confocal images of the sections were collected using a Leica SPE and processed using the Leica application suite X software (LASX) and Adobe Photoshop CS4. From the other group, guts were dissected and used for whole gut imaging with a Nikon SMZ18 microscope and Luxendo GmbH (Heidelberg) 25× MuVi-SPIM. In general, image processing was conducted with Fiji (Schindelin et al., 2012; Schneider et al., 2012). Imaris software was used for 3D reconstructions. Surfaces of clones and the related movies were rendered with Chimera from UCSF (Pettersen et al., 2004). Raw data movies of Gaudí*^LxBBW^* were rendered with Vaa3d (Peng et al., 2014). Quantification of clone size was conducted by thresholding for nuclei and measuring clone volume with the BoneJ Command ‘Particle Analyser’ (Doube et al., 2010). This clone size was divided by a standard size of cell nuclei found in the data (4.4 µm³) and rounded down to next full number. Subsequently, clones consisting of no cells were discarded as artefacts. GraphPad Prism v.7 (www.graphpad.com) was used for statistical analysis and visualization of the data.

### Immunohistochemistry and H&E staining

Immunostaining and Haematoxylin and Eosin (H&E) staining were performed as described previously (Inoue and Wittbrodt, 2011), using the following primary antibodies (all at 1:500): chicken anti-EGFP (Life Technologies, A10262), rabbit anti-phospho H3 (Upstate Biotechnology, 06-570) and rabbit anti-caspase 3 (Abcam, ab13847). Nuclear DNA was stained with DAPI (Sigma, D9564) or DRAQ5 (Thermo Fisher Scientific, 62251; Smith et al., 2004).

### Phylogenetic analyses

Sequences of various vertebrate LGR proteins were retrieved from NCBI: *Danio rerio* LGR4 (accession no. E7FE13), *Danio rerio* LGR6 (P0DM44), Human LGR4 (AAH33039), Human LGR5 (AAH96325), Human LGR6 (AAH47905), Human LGR7 (AAG17167), mouse LGR4 (NP_766259), mouse LGR5 (NP_034325), mouse LGR6 (NP_001028581), *Oryzias latipes* LGR4 (BAM29306), *Oryzias latipes* LGR5 (predicted, XP_004085697), *Oryzias latipes* LGR6 (BAM29305), *Xenopus laevis* LGR5a (ADK66918) and *Xenopus laevis* LGR5b (ADZ55458). BLAST at the ENSEMBL (http://www.ensembl.org/info/about/species.html) confirmed identity of medaka *lgr5* (Oryzias latipes, scaffold2947: 4002-5617). Alignments were created with ClustalW and phylogenetic relationships were deduced after alignment of protein sequences with Geneious (v.8.1.6) following a neighbour-joining approach.
